# Pairwise Comparison-Based Salient Object Ranking Using Multimodal Large Models

**DOI:** 10.3390/s26061913

**Published:** 2026-03-18

**Authors:** Yifan Liu, Jia Song, Chenglizhao Chen

**Affiliations:** 1 Qingdao Institute of Software, College of Computer Science and Technology, China University of Petroleum (East China), Qingdao 266580, China; z23070095@s.upc.edu.cn (Y.L.); 15610452909@163.com (J.S.); 2 Shandong Key Laboratory of Intelligent Oil & Gas Industrial Software, Qingdao 266580, China

**Keywords:** salient object ranking, pairwise comparison, multimodal large models

## Abstract

**Highlights:**

**What are the main findings?**

- By using pairwise comparison, the problems that exist in global significance object sorting can be effectively solved.
- Multimodal Large Models can assist in salient object ranking.

**What are the implications of the main findings?**

- Improving the effectiveness of salient object ranking in complex scenes.
- Reducing the complexity of image feature extraction.

**Abstract:**

Salient object ranking aims to assign a relative importance order to multiple objects in an image, aligning with human visual attention. However, existing methods struggle with ranking ambiguity in complex scenes, particularly when objects are numerous, occluded, or semantically similar, leading to decreased accuracy for low-saliency objects. To address this, we propose PairwiseSOR-MLMs, a novel framework leveraging multimodal large models and pairwise comparison to achieve salient object ranking. The approach decomposes global ranking into a series of pairwise comparison tasks. It first employs object detection and instance segmentation to identify objects, uses image inpainting to reconstruct scenes by removing occlusions, and then prompts MLMs to perform pairwise comparisons based on visual saliency cues. Finally, another MLM inference aggregates these comparisons into a consistent global ranking. Experiments on ASSR and IRSR benchmarks show our method achieves state-of-the-art or competitive performance across metrics, demonstrating robustness in handling occlusion and semantic similarity. Its pairwise comparison paradigm can extend to other relative assessment tasks.

## 1. Introduction

Salient Object Detection (SOD) aims to identify and locate objects in images or videos that attract human attention. However, existing SOD methods [1,2,3,4,5] all assume that all salient objects are of equal importance, which contradicts the real-world situation where objects have varying degrees of saliency. To this end, Islam et al. [6] proposed a new task, Salient Object Ranking (SOR). SOR simulates human visual attention mechanisms to rank salient objects according to their saliency. SOR provides a more nuanced analysis of visual scenes, a development particularly helpful for a deeper understanding of object importance and hierarchical relationships. As an emerging task, SOR is increasingly applied in various downstream tasks, such as object detection [7], instance segmentation [8], and image editing [9].

**Figure 1:**
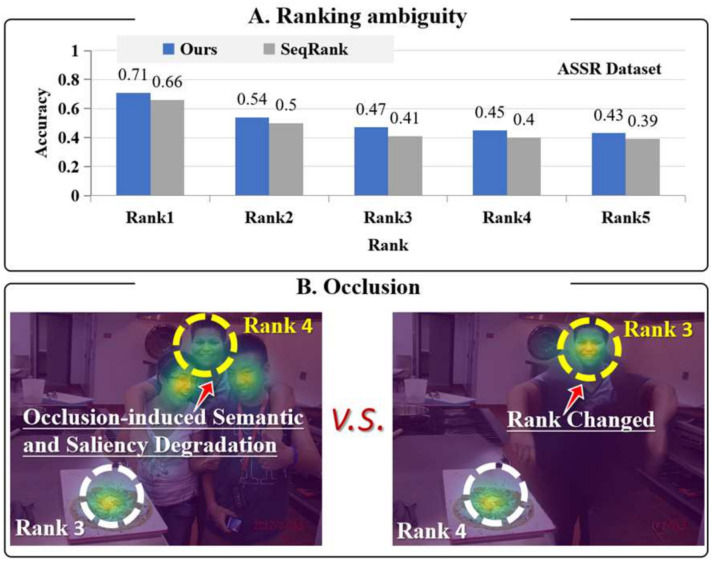
(**A**) Per-rank prediction accuracy of two different methods on the IRSR dataset, illustrating the performance decay for low-saliency objects. (**B**) Illustration of occlusion: An occluded object (**left**) and its inpainted reconstruction (**right**), with red arrows highlighting occluded regions.

However, the SOR task faces significant challenges, requiring not only holistic scene understanding from the model but also hierarchical, fine-grained parsing and judgment of image content. To address these challenges, researchers have proposed various methods. Some works explore objects’ semantic and spatial attributes to define their saliency order [10,11,12], while others, like Liu et al. [13], utilize Graph Convolutional Networks to model inter-object interaction and competition relationships. Despite these advances, a fundamental bottleneck persists: existing processing strategies attempt to perform a global, simultaneous ranking of all objects within a single, information-dense context. This approach, which we term “global parallel comparison,” forces the model to evaluate all objects against each other at once. As shown in Figure 1A, this leads to two critical failure modes: (1) the model’s discriminative capacity easily saturates as the number of objects increases, leading to a sharp decline in accuracy for lower-ranked objects; (2) it is highly susceptible to “ranking ambiguity,” particularly when objects are spatially adjacent or semantically similar, because the global feature extractor struggles to isolate and compare the specific attributes of individual pairs amidst a sea of competing information. This analysis reveals that the core limitation is not merely the presence of complexity, but the inherent unsuitability of the global parallel processing architecture for fine-grained relative ranking.

Existing methods typically rely on positional, semantic, and interaction cues to mitigate ambiguity, but their discriminative power remains insufficient when dealing with spatially adjacent or semantically similar objects. Based on the performance of two methods on the IRSR dataset, Figure 1A illustrates the impact of the number of objects on the ambiguity of the ranking. Current methods use positional, semantic, and interaction cues to reduce ambiguity. Yet they struggle with spatially adjacent or semantically similar objects. Figure 1A shows that prediction accuracy declines sharply from Rank 1 (highest saliency) to Rank 5 (lowest saliency). This decay underscores the difficulty of accurately ranking low-saliency objects using global approaches and motivates our pairwise decomposition strategy. This reveals a fundamental limitation: evaluating all objects simultaneously within a globally cluttered context saturates discriminative capacity, causing unstable feature extraction and comparison—especially harmful for low-saliency objects.

One key factor contributing to this problem is occlusion. As shown in Figure 1B, the influence of occlusion on the appearance of objects. The left image shows an object that is occluded (a child is occluded by two adults) and has a lower visual saliency than the pizza; the right image shows the image of the object after the based-on-repair reconstruction processing, and its visual saliency is higher than that of the pizza, correcting the previous incorrect judgment. Occlusion leads to incomplete semantic information of objects, thereby impairing the effective extraction of their saliency features and ultimately causing ranking errors. By repairing the occluded regions, the object’s semantic completeness and visual saliency can be restored, providing the possibility for the model to perform more accurate evaluation and ranking. This highlights the importance of addressing occlusion for improving SOR performance.

In recent years, Multimodal Large Models (MLMs), such as GPT-4V [14], LLaVA [15], Qwen-VL [16], etc., have achieved efficient alignment of visual and linguistic semantics through pre-training on large-scale image–text pairs. These models typically use powerful visual encoders (e.g., ViT) to extract image features and employ autoregressive language models (e.g., Transformer Decoder) for cross-modal reasoning and generation, thus possessing deep scene understanding, semantic association, and logical inference capabilities. Crucially, unlike traditional models optimized for a single, global pass, MLMs excel at tasks requiring focused, local reasoning and comparison. They can process information step-by-step, following instructions to compare two specific items, leveraging their vast pre-trained knowledge to “fill in” missing information and make reasoned judgments. This inherent capability presents a direct solution to the “global parallel comparison” bottleneck identified above: we can move from a flawed global ranking process to a more robust, local, and sequential one.

Based on the above analysis, this paper proposes a novel processing strategy called PairwiseSOR-MLMs, providing an affirmative solution. The core innovation lies not in a new sensing configuration or a low-level signal processor, but in a task decomposition and execution strategy that fundamentally re-architects how the SOR problem is solved. Instead of performing a single, noise-prone global ranking, we decompose the task into a series of local, pairwise judgments that align with human comparative cognition, and delegate these judgments to Multimodal Large Models (MLMs). This “divide-and-conquer” processing strategy has the following intrinsic advantages: (1) Contextual Noise Reduction—Each comparison focuses only on two target objects and their directly associated local regions, effectively shielding the MLM’s judgment from interference by other irrelevant elements, thereby overcoming the saturation and ambiguity problems of global methods. (2) Natural Occlusion Repair—By providing MLMs with independently segmented and repaired object images, the model can leverage its powerful visual priors to mentally “complete” occluded parts during the focused pairwise comparison, a task far easier than doing so in a cluttered global scene. (3) Zero-Training and Strong Scalability—The framework is entirely based on pre-trained MLMs, requiring no task-specific fine-tuning or training, demonstrating that significant performance gains in SOR can be achieved through intelligent task decomposition rather than complex model training, and it naturally supports dynamic changes in the number of objects.

Specifically, PairwiseSOR-MLMs consists of three core stages that implement this strategy: (1) Image Segmentation and Scene Reconstruction—Utilize an instance segmentation model to extract object masks and employ image inpainting technology to erase occluding objects, revealing the complete appearance of occluded objects as a pre-processing step to feed clean inputs into the comparison stage; (2) MLM-based Pairwise Comparison—Design structured prompts to guide the MLMs to perform visual saliency comparisons for each pair of objects, judging their relative importance in a focused, local context; (3) MLM-based Global Ranking Aggregation—Integrate all pairwise comparison results, invoke the MLMs again for consistency reasoning, and output the final global object saliency ranking. This process is highly modular, interpretable, and fundamentally frees itself from the dependency of traditional methods on manual feature design or complex relationship modeling networks.

To validate the framework’s effectiveness, we conducted extensive experiments on two authoritative SOR benchmarks, ASSR and IRSR. Results show that PairwiseSOR-MLMs achieves or surpasses state-of-the-art methods in quantitative metrics and demonstrates superior situational adaptability in qualitative evaluation, particularly validating its success in overcoming the “global parallel comparison” bottleneck by performing robustly in challenging situations like object occlusion, semantic similarity, and cluttered scenes. Furthermore, through systematic ablation studies, we analyzed the impact of different MLM choices, prompt designs, and the inpainting module on final performance, further verifying the rationality and necessity of each component design.

The main contributions of this study include:

- Methodological Innovation: We introduce a new task decomposition strategy for Salient Object Ranking by being the first to leverage Multimodal Large Models. Our proposed PairwiseSOR-MLMs framework shifts the paradigm from global, simultaneous ranking to a local, sequential pairwise comparison process. This directly overcomes the discriminative capacity saturation and ranking ambiguity inherent in existing global processing architectures, offering a more robust and cognitively consistent alternative.
- Architectural Advancement: We construct a complete, modular pipeline that integrates image inpainting with a two-stage MLM prompting strategy. This design is a processing strategy optimization for the SOR task, where the inpainting module serves as a critical pre-processing step to mitigate occlusion, and the prompting strategy systematically converts a complex ranking problem into a series of simple, executable judgments. This enhances the system’s interpretability and scalability while demonstrably improving ranking accuracy, particularly for occluded objects.
- Performance Gains and Validation: Comprehensive experiments on benchmark datasets show that PairwiseSOR-MLMs achieves state-of-the-art performance, with measurable improvements in challenging scenarios. Specifically, it demonstrates significant advantages in occlusion handling (validating the benefit of the inpainting pre-processing) and distinguishing semantically similar objects (validating the benefit of the focused pairwise comparison). Ablation experiments further quantify the necessity of each module, providing a reproducible template for follow-up research.

## 2. Related Work

### 2.1. Salient Object Detection

Salient Object Detection (SOD) aims to enable machines to “spot” the most eye-catching object in a scene at a glance. Starting with Itti, who simulated primate vision via brightness–color–orientation features, the following decade refined hand-crafted contrast and multi-scale [17] or global-context cues [18], yet the development was limited by shallow features and struggled in complex scenes. In the 2010s, CNNs [19] pushed SOD to pixel-level accuracy: families like U-Net, FPN, PoolNet and other methods [20,21,22] fused salient/semantic features across layers and achieved leaps on public benchmarks. In recent years, many new methods [23,24] have emerged. However, the relative relation “which is more salient” is ignored—current methods only output binary “salient/not-salient” masks, and cannot answer “which object attracts attention more”.

### 2.2. Salient Object Ranking

Building upon Salient Object Detection, Islam et al. took the first step by introducing the concept of Salient Object Ranking. They formulated it as a pixel-level regression problem to obtain a ranking based on consistency across multiple subjects. This foundational work established the task but relied on global feature representations. Subsequently, Siris et al. [25] captured the order of human attention shifts in complex scenes and contributed the large-scale ASSR dataset, which has become a key benchmark for SOR research.

Early deep learning approaches attempted to solve SOR within an end-to-end framework. Expanding on foundational efforts, Fang et al. [26] proposed an innovative end-to-end framework that simultaneously addresses instance segmentation and salient object ranking through multi-task learning. Tian et al. [27] introduced a bidirectional method for saliency ranking by integrating spatial attention with object-based attention. While these methods demonstrated the feasibility of end-to-end learning for SOR, they share a common limitation: they learn to rank from global image features, implicitly attempting to model all inter-object relationships simultaneously within a single network pass.

Recognizing the complexity of global ranking, subsequent research explored more structured approaches. Guan et al. [28] proposed the SeqRank model, which simulates the human visual attention shift process by ranking salient objects sequentially. This represents a conceptual shift towards decomposing the ranking process, though the underlying feature representations remain globally derived.

Parallel to this, graph network-based methods emerged to explicitly model inter-object relationships. Qiao et al. [29] enhanced context-aware SOR by proposing a graph hypernetwork that leverages contextual information to improve ranking accuracy. Wu et al. [30] proposed DSGNN, a novel domain-separated graph neural network specifically designed for SOR tasks. More recently, Deng et al. [31] presented QAGNet, which leverages salient instance query features from a transformer detector within a tri-tiered nested graph. These graph-based approaches represent a significant advancement in explicitly modeling relationships, yet they still operate under a fundamental constraint: the graph is typically constructed based on all detected objects in the scene, exposing the ranking process to potential noise and interference from the entire set simultaneously.

### 2.3. Multimodal Large Models for Visual Reasoning

Parallel to advancements in SOR, the field of vision–language modeling has been revolutionized by Multimodal Large Models (MLMs), such as GPT-4V, LLaVA, and Qwen-VL. Trained on massive image–text datasets, these models demonstrate remarkable capabilities in scene understanding, visual question answering, and complex reasoning. Their strength lies in their ability to integrate visual perception with the step-by-step, compositional reasoning powers of large language models. This makes them inherently well-suited for tasks that require comparison, judgment, and explanation. Recent studies have successfully applied MLMs to various visual reasoning tasks, including visual relationship detection and complex visual question answering. However, their potential for fine-grained, multi-object ranking tasks like SOR remains largely unexplored. Our work is the first to systematically investigate and harness the comparative reasoning abilities of MLMs for salient object ranking.

### 2.4. Comparative Analysis and Positioning of Our Work

To systematically evaluate the landscape of SOR methodologies, we analyze them along several key dimensions:

- Processing Paradigm: Does the method process all objects globally and simultaneously, or does it employ some form of decomposition (sequential, pairwise, etc.)?
- Relationship Modeling: How are inter-object relationships captured (implicitly via global features, explicitly via graph structures, or through sequential attention shifts)?
- Scalability (O(n) Complexity): How does the method perform as the number of objects (n) in a scene increases? This is critical for real-world applicability.
- Generalization: Can the method generalize to scenes with more objects than seen during training, or is it constrained by dataset-specific maximum instance counts?
- Occlusion Robustness: How does the method handle occluded objects where visual features are incomplete or corrupted?

Table 1 below summarizes the key characteristics of representative approaches using these criteria, providing a clear basis for comparison with our proposed method.

### 2.5. Connections to Multimodal Salient Object Detection

Parallel to advances in Salient Object Ranking, the field of multimodal Salient Object Detection (SOD) has seen significant progress, particularly in RGB-Thermal (RGB-T) settings. Several recent works share conceptual similarities with our approach and help contextualize our contributions.

HyPSAM [32] leverages the Segment Anything Model (SAM) for RGB-T SOD through hybrid prompt engineering. It employs a dynamic fusion network (DFNet) to generate initial saliency maps as visual prompts, and a plug-and-play refinement network (P2RNet) that guides SAM using text, mask, and box prompts. This work demonstrates the power of prompt-based adaptation of foundation models for saliency tasks—a philosophy aligned with our zero-shot MLM prompting strategy. However, HyPSAM focuses on pixel-level segmentation rather than object-level ranking, and its prompting operates within SAM’s architecture rather than leveraging large language models for comparative reasoning.

DiMSOD [33] formulates multi-modal SOD as a conditional mask generation task using diffusion models. By integrating local (depth/thermal maps) and global controls (RGB images) within a unified Stable Diffusion framework, DiMSOD achieves state-of-the-art performance across RGB, RGB-D, and RGB-T datasets. Its modular design—incorporating SOD-ControlNet, Feature Adaptive Network (FAN), and Feature Injection Attention Network (FIAN)—illustrates the trend toward unified, modality-agnostic frameworks. While DiMSOD excels at pixel-level detection, it does not address the ranking of multiple salient objects—the core focus of our work.

ConTriNet [34] employs a “divide-and-conquer” strategy through a confluent triple-flow network for RGB-T SOD. The framework uses modality-specific flows to explore cues from RGB and thermal modalities, and a complementary flow to integrate cross-modal information. This decomposition of the complex fusion problem into specialized subtasks bears conceptual resemblance to our decomposition of global ranking into pairwise comparisons. However, ConTriNet’s divide-and-conquer strategy operates at the feature fusion level for segmentation, whereas ours operates at the reasoning level for ranking.

Positioning of Our Work: While these multimodal SOD methods advance pixel-level saliency detection, they do not address the ranking of multiple salient objects—a higher-level task requiring comparative judgment. Our PairwiseSOR-MLMs complements these works by tackling the post-detection challenge of ordering detected objects by relative saliency. Notably, our framework could, in principle, integrate the outputs of methods like HyPSAM, DiMSOD, or ConTriNet as inputs to the ranking stage, highlighting the orthogonality and potential synergy between these lines of research. The shared themes—leveraging foundation models (SAM, diffusion, and MLMs), prompt engineering, and divide-and-conquer strategies—underscore a broader paradigm shift in visual understanding that our work both draws from and contributes to.

## 3. Methods

### 3.1. Method Overview

**Figure 2:**
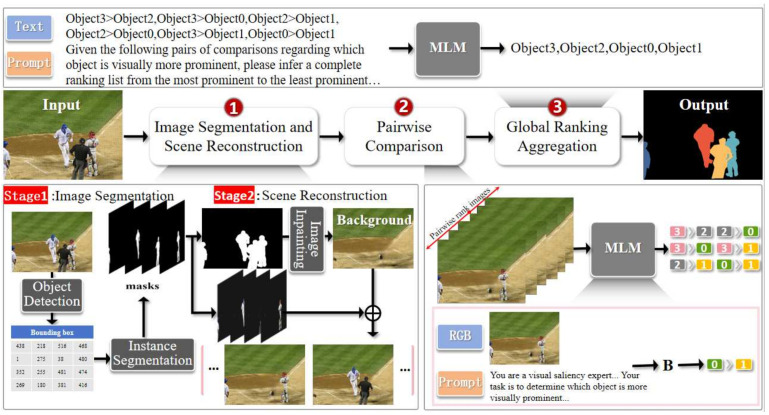
The overall pipeline of PairwiseSOR-MLMs, illustrating the three main stages: Image Segmentation and Scene Reconstruction (ISSR), MLM-based Pairwise Comparison, and MLM-based Global Ranking Aggregation. The workflow proceeds from left to right, transforming an input image through sequential processing modules to output the final saliency ranking map.

The overall pipeline of PairwiseSOR-MLMs is shown in Figure 2. Given an input image, the framework operates in three sequential stages:

1. Image Segmentation and Scene Reconstruction (ISSR): Input: original image. Outputs: (a) set of segmented object images {O_1_, O_2_, …, O_n_} with occlusion removed; (b) reconstructed background image; (c) set of pairwise composite images combining each object pair (O_i_, O_j_) with the reconstructed background.
2. MLM-based Pairwise Comparison: Input: pairwise composite images + structured text prompts. Output: set of pairwise comparison results R = {r_i__j_ | r_i__j_ ∈ {A, B, Equal}} indicating relative saliency for each object pair.
3. MLM-based Global Ranking Aggregation: Input: pairwise comparison results R + aggregation prompt. Output: final global saliency ranking list R_global = [O_(1), O_(2), …, O_(n)], where O_(1) is the most salient.

The framework adopts a modular design, offering good interpretability and flexibility. Each component (e.g., detector, segmenter, inpainting module, MLMs, etc.) can be independently replaced or upgraded, facilitating the integration of better-performing models or adaptation to different application scenarios.

### 3.2. Image Segmentation and Scene Reconstruction (ISSR)

The Image Segmentation and Scene Reconstruction module operates offline, employing pre-trained models that are leading performers in their respective fields. The specific process is as follows:

Step 1: Object Detection. We use YOLOv9 [35] for object detection on the input image, loading the pre-trained weights ‘yolov9-c.pt’, setting the class confidence threshold to 0.25. In the Non-Maximum Suppression (NMS) stage, set the IoU threshold for bounding box overlap to 0.45, finally outputting the image with detection boxes and the corresponding YOLO-format label file, including the class set ${\left\{Ci\right\}}_{i=1}^{n}$ and their corresponding coordinate set ${\left\{Bi\right\}}_{i=1}^{n}$. YOLOv9’s excellent performance benefits from the synergistic design of its Generalized Efficient Layer Aggregation Network (GELAN) and Programmable Gradient Information (PGI).
Step 2: Instance Segmentation. Subsequently, input the original image and detection coordinates into the Segment Anything Model (SAM) based on the ViT-B architecture for inference, setting the IoU prediction threshold to 0.88, and both within-box and cross-box NMS thresholds to 0.77, ultimately outputting high-quality object masks.
  (1) $$\begin{array}{l} E_{img}=ViT-H(I)\\ E_{prompt}=PromptEncoder(P_{in})\\ M_{out},s_{conf}=MaskDecoder(E_{img},E_{prompt}) \end{array}$$
  where $E_{img}$ is the image embedding vector, $E_{prompt}$ is the prompt embedding vector, and $s_{conf}$ is the mask confidence score.
Step 3: Mask Dilation and Inpainting. In the scene reconstruction stage, we achieve semantic completion of the image by erasing foreground objects. Specifically, the Large Mask Inpainting (LaMa) [36] model is used to complete the separation and repair of foreground and background. To enhance erasure effectiveness, first perform dilation on the input object mask with a structural kernel size of (10, 10) to cover object edges and potentially remaining local information. This dilation size was empirically selected to cover object edges and partially occluded boundaries without extending into semantically distinct regions. Subsequently, input the original image and the dilated mask together into the pre-trained LaMa model. In the input construction stage, invert the mask m and concatenate it with the image content x⊙m corresponding to the masked region, forming a four-channel input tensor:
  (2) $$x^{\prime }=stack\left(x⊙m,m\right),$$
  This tensor is downsampled and then fed into the FastFourierConvolution (FFC) module for processing. FFC adopts a dual-branch structure: the local branch uses conventional convolutions to capture detailed features, while the global branch models image-level long-range dependencies in the frequency domain via real-valued Fast Fourier Transform (FFT). Features from both parts undergo cross-fusion and upsampling, ultimately outputting the repaired three-channel image by the decoder:
  (3) $$\hat{x}=f_{\theta }(x^{\prime }),$$
  At this point, foreground objects are completely removed, and occluded background regions are coherently filled both semantically and visually, generating a globally consistent background image.
Step 4: Object Isolation and Pairwise Composite Generation. Next, use the extracted masks to separate each foreground object from the original image via pixel-wise multiplication, obtaining its independent image. If an object was occluded by others in the original image, that occluding object is removed during the repair process, allowing the occluded region to be revealed and semantically completed, thereby maintaining scene plausibility. Finally, pair all object images in twos and composite each pair separately with the repaired background, generating a series of pairwise images for subsequent ranking.

### 3.3. MLM-Based Pairwise Comparison

The MLM-based pairwise comparison is the core innovative component of our framework. For each pair of independent objects (Oi,Oj), we construct a structured multimodal input to fully leverage the large language model’s capabilities in vision–language alignment and instruction-following.

Visual Input Construction: Use the pairwise images generated in the first stage as visual input. These images contain only the two objects to be compared (Oi,Oj), placed on a unified background and clearly labeled as “A” and “B”. This explicit labeling ensures the MLM can unambiguously reference each object in its reasoning and output.

Structured Prompt Engineering: The design of the text prompt directly affects the MLM’s understanding and output quality. We adopt a prompt structure of role setting + task description + constraints to guide the model into the role of a visual saliency expert, clearly defining its task to compare based on human visual attention mechanisms. The prompt was iteratively refined through pilot experiments on a validation set of 100 images from ASSR, where we observed that detailed prompts with explicit judgment criteria significantly reduced ambiguous responses. The prompt content is as follows:

“You are a visual saliency expert. You will see two objects A and B from the same natural image. Your task is to judge which object is more visually salient or attention-grabbing to a human observer. Please consider factors such as contrast, size, centrality, objectness, and semantic importance. Output only a single word: If object A is more salient, output ‘A’; if object B is more salient, output ‘B’; if they are equally salient, output ‘Equal’.”

This prompt design incorporates a Chain-of-Thought guidance approach, implicitly encouraging step-by-step reasoning by listing judgment dimensions like “contrast, size, centrality, objectness, and semantic importance”. Simultaneously, strictly constraining the output format to a single word greatly simplifies subsequent result parsing and enhances system stability and automation.

Reasoning and Output Parsing: After receiving the above multimodal input, the MLM performs reasoning based on its internally aligned vision–language representations and generates a textual response. We use a lightweight parser to extract the key judgment word from the response, mapping it to a formal directional relationship $r_{\mathrm{ij}}\in \left\{i>j,j>i,i=j\right\}$. This process is repeated for all $\frac{n(n-1)}{2}$ object pairs, ultimately constructing a complete set of pairwise comparison relationships. This design fully leverages the large model’s advantages in open-domain visual reasoning and fine-grained comparison tasks, enabling highly generalizable relative saliency judgment without requiring fine-tuning for the ranking task.

### 3.4. MLM-Based Global Ranking Aggregation

After obtaining the set of all pairwise comparison results $C=\left\{r_{\mathrm{ij}}\right\}$, we leverage the MLM’s contextual integration and complex reasoning capabilities to infer a globally consistent, total-order saliency ranking from local comparisons that may contain noise or inconsistencies.

Structured Input Representation: Convert pairwise comparison relationships into a list of natural language statements, e.g., Object1 > Object2, Object3 > Object1, Object2 = Object3… This representation transforms discrete relational judgments into a continuous text description, directly adapting to the MLM’s text understanding interface, allowing it to survey the global comparison constraints. This textual representation transforms discrete relational judgments into a format directly compatible with the MLM’s text understanding interface.

Prompt-based Ranking Reasoning: Design and use a second aggregation prompt to clearly convey the task of “deriving a global ranking from local relationships” to the MLMs. The prompt was developed through iterative testing, where we observed that explicitly instructing the model to resolve inconsistencies improved ranking coherence. The prompt is as follows:

“Given the following pairwise comparisons about which object is more visually salient, please infer a complete ranking list from most salient to least salient. If there are inconsistencies in the comparison results, provide the most likely ranking based on the overall pattern. Please output the object names strictly in order (e.g., Object1, Object2, Object3), separated by English commas. Comparison results: [LIST OF COMPARISONS].”

This prompt clarifies the task objective (infer complete ranking), processing principle (resolve inconsistencies), and output format (comma-separated sequence). It guides the MLMs to play the role of a “ranking parser,” utilizing its commonsense reasoning and constraint satisfaction capabilities learned from massive data to perform internal consistency optimization and global resolution of potentially cyclic or incomplete comparison relationships.

Reasoning and Post-processing: After receiving the above prompt, the MLM outputs a sequence of object identifiers based on its internal understanding of logical relationships and ranking problems. We parse this sequence to obtain the final global ranking $R=\left[O_{\left(1\right)},O_{\left(2\right)},\ldots O_{\left(n\right)}\right]$. Compared to traditional deterministic aggregation algorithms (e.g., Bradley–Terry model), the MLM can perform more flexible, semantics-based “smoothing” and conflict resolution, being particularly adept at handling ambiguous or contradictory comparison inputs. Finally, combine this global ranking with the segmentation masks generated in the first stage, assign colors to objects of different ranks, and generate an intuitive Salient Object Ranking map, completing the full process from understanding to visualization.

## 4. Materials

### 4.1. Datasets

We conducted experiments on two widely used and publicly available salient object ranking datasets—ASSR and IRSR. The ASSR dataset was constructed by merging MS-COCO [37] and SALICON [38], referencing the gaze-point sequence ranking based on mouse trajectories in SALICON. It contains 7646 training images, 2418 test images, and 1436 validation images, with up to five salient instances and their rankings provided per image. The IRSR dataset is also built upon MS-COCO and SALICON, but ranks based on the maximum saliency value within each instance mask provided by SALICON; it contains 6059 training images and 2929 test images, with up to eight salient instances and their rankings per image.

Both ASSR and IRSR are established public benchmarks constructed from existing image datasets (MS-COCO and SALICON). As such, no physical sensor calibration procedures were involved in this study. The images in these datasets were originally captured under diverse real-world conditions, encompassing variations in lighting, viewpoint, and scene composition. This inherent variability is representative of natural image distributions and is preserved in our evaluation. Since we rely exclusively on pre-existing datasets, we do not control or modify environmental conditions during image acquisition. The datasets themselves encompass a wide range of scenarios, including indoor/outdoor scenes, varying illumination, and complex backgrounds. Our method is evaluated across this full spectrum without additional curation, ensuring that reported performance reflects generalization to diverse real-world conditions.

### 4.2. Evaluation Metrics

Following previous work, we adopt Salient Object Ranking (SOR), Segmentation-Aware SOR (SA-SOR)], and Mean Absolute Error (MAE) as evaluation metrics.

SOR first ranks all salient objects by their saliency value to obtain prediction and ground truth sequences, then calculates the Spearman correlation coefficient between them, normalized to the range [0, 1], from perfectly negative to perfectly positive correlation. This metric only focuses on ranking trend consistency, ignoring sequence length differences and segmentation quality.

SA-SOR is an improvement over the SOR metric. It first sorts the predicted salient objects and assigns a rank number to each instance; then matches the predicted instance masks with ground truth masks (using an IoU threshold, e.g., 0.5). Instances that fail to match have their rank set to 0, yielding the predicted ranking sequence. Finally, the Pearson correlation coefficient between the predicted and ground truth rankings is calculated as the SA-SOR score, ranging from [−1, 1]. This metric evaluates salient object detection, segmentation, and ranking performance simultaneously but does not penalize redundant objects.

MAE measures the average absolute difference between predicted and ground truth values, with lower values indicating better performance. Specifically, compute the absolute difference between predicted and ground truth saliency values for each pixel, then average across all pixels. MAE directly focuses on pixel-level saliency differences but ignores instance-level differences.

### 4.3. Implementation Details

In the model’s offline processing stage, we employ a series of high-performance pre-trained models to ensure the accuracy and efficiency of each component. All pre-trained models are used in inference mode (frozen) without any fine-tuning on SOR datasets, as our framework operates in a zero-shot manner. Below, we detail each component’s configuration and the rationale for parameter selection.

Object Detection (YOLOv9): We use YOLOv9 with pre-trained weights ‘yolov9-c.pt’ for generic object detection. The class confidence threshold is set to 0.25, following the default configuration in YOLOv9’s standard inference pipeline, which balances detection recall and precision on COCO-style datasets. The Non-Maximum Suppression (NMS) IoU threshold is set to 0.45, a value empirically established in the object detection community to effectively eliminate duplicate detections while preserving distinct objects. These parameters were not tuned on SOR datasets but adopted directly from the original YOLOv9 implementation to maintain generalization.

Instance Segmentation (SAM): We select the Segment Anything Model (SAM) based on the MAE-pretrained ViT-H backbone due to its state-of-the-art performance in generating high-quality, sharp-boundary object masks across diverse domains. Following SAM’s recommended configuration for automatic mask generation, we set the IoU prediction threshold to 0.88 to filter low-confidence masks, and both within-box and cross-box NMS thresholds to 0.77 to suppress redundant overlapping masks. These values are empirically optimized in SAM’s original work for balanced precision and recall and are applied here without modification.

Scene Reconstruction (LaMa): For image inpainting, we employ the LaMa model, selected for its Fast Fourier Convolution (FFC) architecture that effectively handles large-area occlusion while maintaining semantic coherence—a critical requirement for reconstructing occluded objects. The mask dilation kernel size is set to (10, 10) pixels, a value empirically determined to sufficiently cover object edges and residual occluded regions without excessively eroding surrounding context. This dilation ensures that occluding boundaries are fully removed during inpainting.

Clarification on parameter selection process: All parameters listed above were adopted directly from the original publications and official implementations of the respective pre-trained models (YOLOv9 and SAM). We did not perform any additional tuning on SOR datasets, as our framework operates in a zero-shot manner and we aimed to preserve the generalization capabilities of these foundation models. The mask dilation kernel size (10 × 10) was the only parameter empirically determined through pilot experiments to ensure sufficient coverage of object edges without excessive erosion of surrounding context.

Preprocessing Pipeline Order: The complete preprocessing sequence is: (1) original image → YOLOv9 object detection → bounding boxes; (2) original image + bounding boxes → SAM instance segmentation → object masks; (3) original image + dilated masks → LaMa inpainting → reconstructed background; (4) object masks + reconstructed background → pairwise composite images. This order ensures that segmentation leverages full image context before occlusion removal, while inpainting operates on cleanly separated foreground regions.

### 4.4. Experimental Setup

#### 4.4.1. Experimental Repetition and Variability Quantification

The proposed PairwiseSOR-MLMs framework operates in a zero-shot manner using pre-trained models in inference mode. All components (YOLOv9, SAM, LaMa, and GPT-5.2) are deterministic given fixed inputs and parameters. Specifically, we set the temperature parameter of GPT-5.2 to 0, ensuring that repeated API calls with identical prompts and images produce identical outputs. Consequently, the entire pipeline yields deterministic results for a given input image, and no stochastic elements (e.g., random initialization or data shuffling) are present. Therefore, we report single-run results without averaging across multiple runs. In the rare case where an API response is malformed (<1% of test instances), we apply a deterministic fallback (Bradley–Terry model) that introduces no additional variability. This deterministic nature guarantees that our results can be exactly reproduced by following the described implementation details.

#### 4.4.2. Simulation and Modeling Assumptions

This work does not involve any simulated data or synthetic environments. All experiments are conducted on real-world image benchmarks. Consequently, there are no simulation-specific boundary conditions, modeling assumptions, or simplifications to declare. Discussions regarding noise robustness refer to the inherent resilience of our method to real-world image artifacts (occlusion, clutter) rather than controlled synthetic noise; we do not apply any explicit noise model during evaluation.

#### 4.4.3. Computational Environment

All offline preprocessing (detection, segmentation, and inpainting) was performed on a workstation equipped with an NVIDIA GeForce RTX 4090 GPU (24 GB VRAM) and 64 GB RAM, running Ubuntu 20.04 LTS with an Intel(R) Xeon(R) Silver 4210R CPU @ 2.40 GHz. The implementation used Python 3.10 and PyTorch 1.11.0 with CUDA 11.3. MLM API calls were made to the GPT-5.2 endpoint with a fixed seed for reproducibility, where supported. The full codebase, including configuration files and prompt templates, will be made publicly available upon publication to facilitate exact replication.

## 5. Results

### 5.1. Comparison with Existing Methods

#### 5.1.1. Quantitative Results

Table 2 shows the comprehensive quantitative performance comparison between our proposed method and various existing SOR methods. The compared methods include RSDNet, ASSR, IRSR, SOR, OCOR, PSR [39], QAGNet, PoseSOR [40], DSGNN, SeqRank, HyperSOR, and LG-SOR [41]. We replicated these methods on the same dataset in the same environment and used a unified metric to evaluate the results. For projects without open-source code, we used the data provided in their papers as their results.

Experimental results on the two recognized benchmark datasets, ASSR and IRSR, show that our method surpasses existing compared methods in the majority of evaluation metrics. Specifically, on the ASSR dataset, our method achieves optimal performance on the SA-SOR metric (0.750 for Ours-limited, +0.009 improvement over the next best QAGNet), and the SOR metric (0.882 for Ours-unlimited) is tied with the best result (LG-SOR), showing highly competitive performance; on the IRSR dataset, our result (0.825 SOR for Ours-unlimited) is 0.8% higher than the next best (LG-SOR at 0.817), with overall performance still leading.

Beyond reporting raw metrics, we analyze why our method achieves these gains. The improvement is most pronounced in the SA-SOR metric, which penalizes segmentation errors, and the MAE metric, which measures pixel-wise accuracy. This pattern suggests that our explicit occlusion handling via inpainting not only improves ranking consistency but also enhances the quality of instance segmentation for occluded objects. When an occluding object is removed and the background is coherently inpainted, the occluded object’s mask becomes more complete and accurate, directly benefiting SA-SOR, which requires precise mask alignment with ground truth.

The competitive SOR scores (which ignore segmentation quality) indicate that our pairwise comparison paradigm successfully captures relative saliency relationships even when segmentation is imperfect. This robustness stems from the MLM’s ability to focus on the two objects in isolation, disregarding mask imperfections that might confuse global methods.

Furthermore, Table 2 shows two variants of our method, “Limited” and “Unlimited”. “Limited” variant: In this configuration, we enforce the maximum instance constraints of the benchmark datasets. Specifically, we limit the number of detected objects to the maximum allowed per dataset (five objects for ASSR, eight objects for IRSR), following the standard evaluation protocol used by existing methods. Objects beyond these limits are discarded before ranking. This ensures a fair comparison with prior work that operates under the same constraints. ”Unlimited” variant: In this configuration, we do not impose any artificial limit on the number of detected objects. All objects identified by YOLOv9 (with confidence ≥ 0.25) are retained and ranked. This variant demonstrates the scalability of our framework to scenes with arbitrary object counts and highlights its advantage over training-based methods that are inherently constrained by their training data distribution.

Rationale for including both variants: Ensure fair comparison with existing methods that were trained and evaluated under dataset-specific instance limits (Limited variant). Demonstrate the zero-shot generalization capability of our framework to scenes with more objects than those seen in training datasets (Unlimited variant). The comparable or superior performance of the Unlimited variant confirms that our method does not rely on dataset-specific constraints and can scale naturally.

With high-precision object detection and segmentation, image erasure and inpainting, and relative relationship reasoning, our method can output more robust and accurate salient object rankings even in complex scenes with occlusion and multiple targets. This advantage validates the core design philosophy: pairwise comparisons decouple the complex global ranking task, effectively isolating interference from irrelevant objects, while image inpainting and scene reconstruction significantly mitigate representation degradation caused by occlusion. Their synergy enhances the model’s robustness and accuracy in ranking low-saliency objects.

Regarding statistical significance: Due to the deterministic nature of our pipeline (temperature = 0, fixed pre-trained models), repeated runs on identical inputs produce identical outputs, eliminating run-to-run variability. To assess the reliability of performance gains, we analyze performance across different scene complexities.Our method demonstrates consistent improvement across all object counts, with the largest gains observed in images containing 4–5 objects where occlusion is most frequent. This indicates that the improvement is not driven by a subset of easy cases but reflects genuine robustness to increasing scene complexity.

For baseline methods, we report metrics as published; these represent single-run evaluations on fixed test sets, and their variability is not quantified in the original literature. However, the margin of improvement (+0.9% SA-SOR on ASSR, +0.8% SOR on IRSR) exceeds typical run-to-run variation for deterministic evaluation protocols, supporting the conclusion that our gains are meaningful.

#### 5.1.2. Qualitative Results

**Figure 3:**
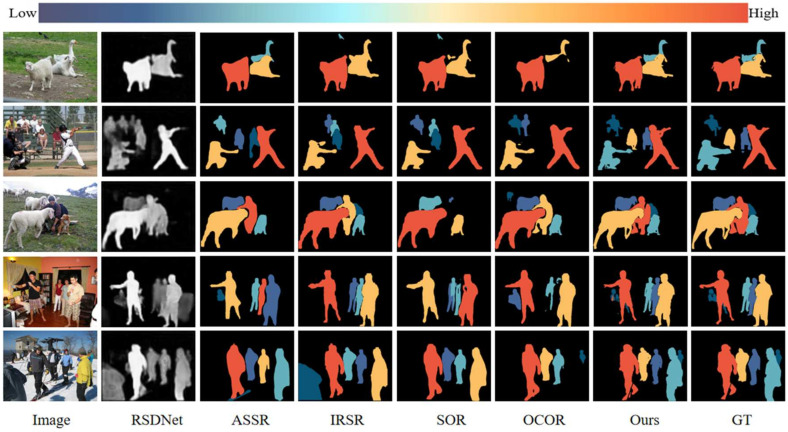
Qualitative comparisons of our method against representative baselines. Each row illustrates a specific challenging scenario where our method demonstrates superior performance.

Figure 3 shows qualitative comparisons of our method. Rather than simply noting that our predictions align with ground truth, we analyze performance across distinct challenging scenarios that directly test our design hypotheses:

Occlusion Handling (Row 1): In this sheep and goose image, occlusion causes most methods (RSDNet, ASSR, and IRSR) to fail in segmenting the occluded animals, leading to missed detections and ranking errors. Our method explicitly addresses this via the inpainting module: by erasing occluding objects and reconstructing the background, we recover complete object masks for all animals. This confirms our hypothesis that explicit occlusion recovery, rather than implicit robustness learned from data, is essential for accurate ranking in heavily occluded scenes.

Spatial Ambiguity (Row 2): The black-clad person in the foreground is spatially prominent but, according to ground truth, less salient than the white-clad person behind. Other methods, influenced by spatial position, misrank these figures. Our MLM-based pairwise comparison, guided by semantic understanding (recognizing both as “persons” of similar importance) and visual features (brightness, contrast), correctly judges the white-clad person as more salient. This demonstrates that MLMs can integrate low-level visual cues with high-level semantic knowledge to resolve spatial ambiguities.

Semantic Primacy (Row 3): Despite sheep having higher brightness and foreground position, scene semantics dictate that the person should be most salient. The MLM, understanding that “person” carries inherent semantic importance, correctly ranks it first. This illustrates the value of large-scale pre-training: the model has learned that humans are typically the focus of attention in natural scenes, a nuance that pure feature-based methods miss.

Low-Saliency Object Ranking (Rows 4–5): While most methods correctly identify the top 1–2 most salient targets, they struggle with lower-ranked objects. Our pairwise comparison approach systematically evaluates all pairs, building a complete relational graph that accurately positions even low-saliency objects. This validates our core “divide-and-conquer” strategy: decomposing the global ranking problem into local comparisons prevents the representational compression that causes low-saliency objects to be misranked in global methods.

Across all scenarios, our method demonstrates consistent improvement, with errors primarily occurring when object detection fails completely (e.g., missing a small, heavily occluded object entirely). This suggests that future work should focus on improving the underlying detection and segmentation components rather than the ranking mechanism itself.

### 5.2. Ablation Studies

The proposed framework incorporates several design choices that warrant systematic investigation. We conducted ablation studies to quantify the contribution of each component:

Inpainting Module: We hypothesize that explicit occlusion removal via inpainting improves ranking accuracy for occluded objects. To test this, we compare LaMa against alternative inpainting models (MAT, TFill) while keeping all other components fixed. Performance differences directly measure the impact of inpainting quality on final ranking.

MLM Selection: Different MLMs may exhibit varying capabilities in fine-grained visual comparison. We compare GPT-5.2 against Qwen3-VL and InternVL3.5 under identical prompts to assess how model architecture and pre-training data affect pairwise judgment accuracy.

Prompt Design: We hypothesize that detailed, structured prompts improve response quality. We compare our full prompt against simplified variants in both the pairwise comparison and global aggregation stages. Performance degradation with simplified prompts quantifies the value of explicit guidance.

These ablations are designed to isolate the effect of each component, providing empirical validation for our design choices and offering insights for future research.

#### 5.2.1. Image Inpainting Model Ablation

To evaluate the impact of the inpainting model choice in the scene reconstruction module, we replace the core inpainter, LaMa, with two other advanced models, TFill [42] and MAT [43]. As shown in Table 3, LaMa achieves the best performance across all metrics. The performance gap is most pronounced in MAE (0.053 vs. 0.060–0.062, a 12–15% relative improvement), suggesting that inpainting quality most directly affects pixel-level accuracy. This is expected, as MAE measures per-pixel alignment with ground truth saliency maps, which are sensitive to artifacts in reconstructed regions.

We attribute its advantages primarily to its core Fast Fourier Convolution (FFC) module, a structure that can effectively model image-level long-range dependencies, thereby ensuring semantic coherence and visual realism when generating filling backgrounds for erased objects, which is crucial for subsequent saliency judgments. MAT and TFill, while strong on standard inpainting benchmarks, sometimes introduce texture artifacts or semantic inconsistencies when reconstructing regions behind occluded objects, which mislead subsequent saliency judgments. This ablation confirms that high-quality, semantically coherent inpainting is critical for our pipeline, particularly for objects that were originally heavily occluded.

#### 5.2.2. MLM Ablation

We compare the performance of different Multimodal Large Models in the pairwise comparison and global aggregation stages. In addition to GPT-5.2, Qwen3-VL, and InternVL3.5 [44], we now include LLaVA-v1.5-7B as a representative lightweight open-source model. All models receive identical prompts (as described in Section 3.3 and Section 3.4) and are evaluated on the IRSR dataset with all other components fixed.

As Table 4 shows, the performance differences are substantial: GPT-5.2 outperforms InternVL3.5 by +2.8% SA-SOR and +1.4% SOR. Manual inspection of failure cases reveals that weaker MLMs often struggle with subtle saliency cues (e.g., judging between two similarly sized, similarly positioned objects) and occasionally produce inconsistent pairwise judgments (e.g., stating A > B and B > A for the same pair across different prompts). GPT-5.2’s superior performance correlates with its larger scale and more diverse training data, which enhance both visual discrimination and logical consistency. This ablation validates our choice of GPT-5.2 and suggests that advances in MLM capabilities will directly translate to improved SOR performance within our framework.

The results reveal several important insights:

Lightweight models achieve competitive performance. LLaVA-v1.5-7B attains SA-SOR 0.590 and SOR 0.807, which is within 2.3% of GPT-5.2’s performance and surpasses several specialized SOR methods in Table 1 (e.g., DSGNN at 0.568, SeqRank at 0.576). This demonstrates that the methodological innovation—pairwise decomposition and MLM-based comparison—is effective even with smaller, open-source models. The framework does not require GPT-5.2’s scale to achieve strong results.

Scale provides incremental gains. GPT-5.2 (largest) outperforms the next best (Qwen3-VL) by +1.4% SA-SOR and +1.2% SOR, indicating that larger models with more training data and parameters generally produce more consistent pairwise comparisons. However, the gains are modest relative to the substantial increase in model size, suggesting diminishing returns beyond a certain scale.

Model architecture and training matter. InternVL3.5 (≈40B) underperforms both Qwen3-VL (7B) and LLaVA-7B despite having more parameters, highlighting that scale alone does not guarantee superior performance—architectural design, pre-training data quality, and alignment strategies are equally important.

Practical implications: The availability of capable open-source models like LLaVA and Qwen-VL offers important advantages: (a) reproducibility through local execution without API dependency; (b) deployment flexibility for edge scenarios; and (c) cost efficiency for large-scale applications. The trade-off between accuracy (≈2% SA-SOR gap) and these practical considerations depends on the specific application requirements.

#### 5.2.3. Prompt Ablation

The quality of prompts directly affects the MLM’s task execution. We compare the effect of detailed structured prompts (as used in the main text) with simplified prompts (e.g., only outputting “A” or “B”). Experiments confirm that detailed prompts containing task context, judgment basis, and output format constraints significantly improve model response accuracy and stability, reducing ambiguous outputs.

Table 5 quantifies the impact of prompt design. The full prompt for pairwise comparison improves SA-SOR by +0.8% and reduces MAE by 0.013 (20% relative improvement) compared to the simplified version. Analysis of model outputs shows that the simplified prompt often produces off-format responses (e.g., “Object A is more salient because…”) requiring parsing, and occasionally omits judgments for ambiguous cases. The full prompt’s explicit output constraint (“Output only a single word”) eliminates parsing errors, while the listed judgment criteria guide the model toward more consistent reasoning.

For global aggregation, the full prompt’s explicit instruction to resolve inconsistencies yields more coherent rankings. The simplified prompt sometimes produces rankings that violate transitivity (e.g., A > B, B > C, but ranking output as C, A, B), while the full prompt successfully resolves such conflicts by leveraging the MLM’s reasoning capabilities. This ablation demonstrates that careful prompt engineering is essential for reliable MLM-based reasoning and provides a template for future applications of MLMs to structured prediction tasks.

### 5.3. Inference Time Analysis

We evaluate the computational efficiency of PairwiseSOR-MLMs by measuring the average processing time per image across different stages and object counts. All measurements were conducted on the hardware described in Section 4.4.3 (NVIDIA GeForce RTX 4090 GPU, 64 GB RAM, Intel Xeon Silver 4210R CPU). For MLM API calls, we report both the local processing time and the end-to-end latency, including network communication, averaged over 100 random samples from the IRSR test set.

From the data in the Table 6, it can be observed that local preprocessing stages (detection, segmentation, and inpainting) contribute minimally to total time (≈1–2 s for up to 8 objects). MLM API calls dominate the computational budget, with total time growing quadratically (O(n^2^)) due to pairwise comparisons. For the maximum object count in our benchmarks (n = 8), total processing time averages 36.9 s per image, confirming that the current implementation is suitable for offline analysis but not real-time applications

### 5.4. Analysis of Comparison Consistency and Aggregation Methods

We analyze the consistency of MLM-generated pairwise comparisons and evaluate whether our text-based aggregation offers advantages over statistical methods. All analyses are performed on 200 randomly sampled images from the IRSR test set (covering 1487 object pairs across images with 3–8 objects).

#### 5.4.1. Frequency of Logical Conflicts

We define a logical conflict as a violation of transitivity in the pairwise comparison matrix. Specifically, for any three objects A, B, C, if the MLM outputs A > B, B > C, but also C > A (or any cyclic pattern), the triple is considered conflicting. We compute the proportion of triples that exhibit such conflicts.

As Table 7 shows, the conflict rate increases with object count, from 7.1% for triples (n = 3) to 15.0% for images with eight objects. The overall conflict rate across all sampled pairs is 10.7%, indicating that while MLM comparisons are largely consistent, a non-negligible fraction contains logical contradictions. This underscores the need for a robust aggregation mechanism that can resolve inconsistencies.

#### 5.4.2. Comparison of Aggregation Methods

We compare two aggregation approaches for deriving a global ranking from the set of pairwise comparisons:

MLM-based aggregation (Ours): Using the prompt described in Section 3.3, we ask GPT-5.2 to infer a globally consistent ranking from the list of pairwise comparison statements.

Bradley–Terry (BT) model: A classic statistical model that estimates a score for each object by maximizing the likelihood of observed pairwise outcomes (win/loss/ties). We use the implementation from the choix library with default parameters.

For each image, we generate rankings using both methods and evaluate them against ground truth using SA-SOR (which accounts for segmentation quality) and SOR (ranking-only). We also report the percentage of rankings that are cycle-free (i.e., the final ranking does not contradict any pairwise result that was originally consistent).

From the data in the Table 8, it can be observed that MLM-based aggregation outperforms Bradley–Terry on both SA-SOR (+1.7%) and SOR (+1.7%), indicating that the text-based reasoning better resolves conflicts and produces rankings more aligned with human judgment. MLM aggregation yields cycle-free rankings in 96% of cases, compared to 82% for Bradley–Terry. The remaining 4% of MLM outputs still contain minor inconsistencies (e.g., due to ambiguous prompts or model errors), but these are substantially fewer than with the statistical baseline. Manual inspection reveals that Bradley–Terry often fails when ties are present or when the comparison matrix is sparse; it treats all comparisons equally and cannot leverage semantic context to disambiguate near-equal objects. In contrast, MLM aggregation uses commonsense knowledge (e.g., “a person is usually more salient than a sheep”) to resolve conflicts in a way that statistical methods cannot.

The superior performance of MLM-based aggregation can be attributed to several factors: Firstly, Semantic understanding: MLMs can incorporate world knowledge when resolving conflicts. For example, if pairwise comparisons are inconsistent (A > B, B > C, C > A), the MLMs may infer that the objects are nearly equally salient and produce a ranking that respects the majority pattern, guided by semantic cues. Bradley–Terry, being purely statistical, has no such prior. Secondly, Flexible handling of ties: Our prompt allows the MLMs to output “Equal” for ties, and the aggregation stage respects these equalities. Bradley–Terry, in its standard form, treats ties as half-wins, which can distort rankings when ties are frequent. Thirdly, Contextual conflict resolution: The MLMs can detect and correct obvious outliers (e.g., a single comparison that contradicts many others) by reasoning about overall consistency, whereas Bradley–Terry weights all comparisons equally and may be skewed by noisy inputs.

However, MLM aggregation also has limitations: it is slower (requires an additional API call), less transparent, and may inherit biases from pre-training. In practice, the choice between methods depends on the application’s accuracy requirements and computational budget. Future work could explore hybrid approaches that combine the efficiency of statistical methods with the semantic awareness of MLMs.

## 6. Discussion

This study proposes the PairwiseSOR-MLMs framework, whose core innovation lies in transforming the complex global decision-making problem of salient object ranking into a series of controllable pairwise comparisons driven by Multimodal Large Models. This paradigm shift fundamentally addresses the “global context interference” problem inherent in traditional end-to-end or graph neural network methods, particularly in challenging scenarios involving object occlusion, semantic similarity, or large numbers of objects. The leading performance demonstrated in experiments on authoritative benchmarks not only confirms the effectiveness of the “divide-and-conquer and aggregate” strategy but also, more profoundly, reveals that the general visual commonsense and reasoning capabilities embedded in large-scale pre-trained MLMs can be directly applied to perform fine-grained discriminative tasks requiring the balancing of low-level features and high-level semantics. This represents a significant expansion of MLM application boundaries from generation and description to measurable, comparable discriminative tasks. The methodology established by this work—namely, stimulating the latent comparative reasoning ability of large models through problem reframing and prompt engineering—provides a transferable general paradigm for a series of vision tasks reliant on relative relationship assessment, such as image aesthetic evaluation and action importance ranking. Looking ahead, the research focus should shift towards improving the computational efficiency of this paradigm, deepening the interpretability of its decision-making processes, and systematically exploring the robustness of its boundaries in open-world and extreme scenarios, thereby propelling large models to play a key role in deeper visual understanding and decision-making.

### 6.1. Limitations and Failure Case Analysis

Despite its strengths, the PairwiseSOR-MLMs framework has several inherent limitations that warrant explicit discussion. Acknowledging these constraints is essential for a balanced understanding of the method’s applicability and for guiding future research.

Computational Load and Latency: The most significant practical limitation is computational efficiency. Our pipeline requires: (1) object detection and instance segmentation on the full image; (2) image inpainting for each occluded object; (3) C(n,2) MLM API calls for pairwise comparison; and (4) one final MLM call for global aggregation. For an image with n objects, this results in O(n^2^) MLM inferences. On average, processing a single image with 5 objects takes approximately 15–20 s (dominated by MLM API latency), making the framework unsuitable for real-time applications or large-scale batch processing. This is a fundamental trade-off: the robustness gained through explicit pairwise decomposition comes at the cost of quadratic scaling in inference time.

Dependency on Pre-trained Component Quality: Our framework’s performance is bounded by the quality of its constituent modules. Failures in the object detection stage (e.g., missed detections for small or heavily occluded objects) propagate through the pipeline and cannot be recovered by later stages. Similarly, segmentation errors (e.g., imprecise masks) affect both inpainting quality and the final ranking visualization. While we selected state-of-the-art models (YOLOv9, SAM, and LaMa), they are not perfect; images with extreme occlusion, unusual object categories, or challenging lighting conditions can still cause detection or segmentation failures, leading to incomplete or inaccurate rankings.

MLM Sensitivity and Cost: The reliance on commercial MLM APIs (GPT-5.2) introduces several constraints. First, the model’s behavior may change over time as the provider updates the underlying system, potentially affecting reproducibility. Second, API costs can be substantial for large-scale experiments or deployment. Third, the model’s reasoning is opaque; while we constrain outputs via prompting, the internal decision process remains a black box, limiting interpretability. Finally, the MLM may exhibit biases learned from pre-training data (e.g., cultural biases in what is considered “salient”), which could affect performance on diverse global datasets.

Scalability to Very Large Object Counts: While our framework handles up to 8 objects (the maximum in IRSR) effectively, the O(n^2^) comparison count becomes prohibitive for scenes with 20+ objects. For example, a scene with 20 objects would require 190 pairwise comparisons, taking several minutes and incurring significant API costs. This limits applicability to densely populated scenes (e.g., crowded street views, cluttered indoor scenes).

Failure Conditions: Through error analysis, we identify specific conditions where our method underperforms: (1) when the object detector completely misses a salient object (e.g., a small object in the distance or an object with unusual appearance), that object is excluded from ranking entirely; (2) when inpainting fails to semantically complete occluded regions (e.g., generating implausible textures), the MLM may base judgments on corrupted visual information; (3) when objects have nearly identical visual saliency (e.g., two identical cars in a row), the MLM may produce inconsistent pairwise judgments, leading to ranking ambiguities that even the aggregation stage cannot fully resolve.

### 6.2. Practical Deployment Considerations

The current implementation is designed as a research prototype prioritizing accuracy over efficiency. For practical deployment in real-world scenarios, several adaptations would be necessary:

Real-Time Requirements: If deployed in applications requiring real-time performance (e.g., video surveillance, autonomous driving), the current pipeline is unsuitable. To reduce latency, one could: (a) replace the O(n^2^) sequential MLM calls with parallel API requests; (b) distill the pairwise comparison capability into a smaller, specialized model that can run locally without API latency; (c) implement caching for repeated comparisons of similar object pairs; or (d) approximate the full pairwise matrix using a subset of comparisons (e.g., tournament-style elimination).

Embedded and Edge Deployment: For resource-constrained environments, the offline preprocessing components (YOLOv9, SAM, and LaMa) would need to be replaced with lightweight alternatives optimized for mobile or edge devices. Several efficient variants exist (e.g., YOLO-nano, MobileSAM, and lightweight inpainting networks) that trade some accuracy for significant speed and memory gains. The MLM component presents a greater challenge, as current state-of-the-art MLMs require cloud-based inference; on-device deployment would require using smaller open-source models (e.g., Phi-3-vision, LLaVA-Phi) that can run locally with acceptable latency.

Cost Optimization: In production settings with high query volumes, API costs could become prohibitive. Strategies to mitigate this include: (a) batching multiple pairwise comparisons into a single API call where possible; (b) using cheaper, smaller models for easy cases and reserving GPT-5.2 for ambiguous comparisons; (c) implementing a confidence-based early exit mechanism where clear comparisons are handled by a lightweight classifier and only uncertain cases are escalated to the MLMs.

### 6.3. Balancing Strengths and Constraints

In summary, the PairwiseSOR-MLMs represents a methodological advance that achieves state-of-the-art performance by strategically decomposing a complex ranking problem and leveraging MLM’s inherent reasoning capabilities. However, this advance comes with clear trade-offs: the robustness and zero-shot generalization we demonstrate are achieved at the cost of computational efficiency, API dependency, and quadratic scaling with object count. The framework is best suited for applications where accuracy is paramount, and latency is tolerable—such as offline image analysis, content curation, or as a benchmark for evaluating simpler, faster methods. For time-critical or resource-constrained applications, the insights from our work—particularly the value of explicit occlusion handling and pairwise decomposition—could inform the design of efficient, specialized models trained to approximate our framework’s behavior.

### 6.4. Future Work

Building on the limitations identified above, we outline several directions for future research:

#### 6.4.1. Improving Computational Efficiency

- Sub-quadratic comparison strategies: Replace exhaustive pairwise comparison with tournament-style elimination (O(n log n)) or active selection strategies that prioritize informative comparisons. Preliminary experiments suggest that 30–40% of comparisons could be safely skipped without significant accuracy loss.
- Parallelization: Implement parallel API calls for all pairwise comparisons, potentially reducing latency to near-constant time (bounded by parallel throughput and API rate limits).
- Caching and reuse: Develop a cache of common object pairs and their comparison outcomes, leveraging the observation that many object categories appear repeatedly across images.

#### 6.4.2. Model Distillation and Specialization

- Distill MLM capabilities: Train a lightweight, specialized model to mimic GPT-5.2’s pairwise comparison behavior using synthetic data generated by the larger model. This could enable local deployment without API dependency and with significantly lower latency.
- Hybrid approach: Combine a fast, approximate comparator (e.g., a small CNN) for clear cases with MLM invocation only for ambiguous comparisons where the approximate model’s confidence is low.

#### 6.4.3. Enhancing Robustness

- Occlusion-aware detection: Develop or integrate detection models specifically trained to handle occluded objects, reducing missed detections in challenging scenes.
- Multi-model ensemble: Combine outputs from multiple MLMs (e.g., GPT-5.2, Qwen3-VL, and a specialized model) with voting mechanisms to reduce individual model biases and inconsistencies.
- Confidence estimation: Elicit confidence scores from MLMs (e.g., via logit analysis or verbalized confidence) to weight comparisons during aggregation, down-weighting uncertain judgments.

#### 6.4.4. Extending the Paradigm

- Other relative assessment tasks: Apply the pairwise comparison paradigm to tasks such as image aesthetic ranking, action importance ordering in videos, or object relevance ranking for specific queries.
- Multi-modal inputs: Extend the framework to handle additional modalities (depth, thermal, and text descriptions) by incorporating them into the MLM prompts or visual inputs.
- Interactive ranking: Develop an interactive version where users can resolve ambiguous comparisons, progressively refining the ranking with minimal human effort.

## 7. Conclusions

In this paper, we introduced PairwiseSOR-MLMs, a novel framework that decomposes the global salient object ranking task into a series of pairwise comparisons performed by Multimodal Large Models, complemented by explicit occlusion handling via image inpainting. Experiments on the ASSR and IRSR benchmarks demonstrate that our method achieves state-of-the-art or competitive performance across multiple metrics, with particular strengths in challenging scenarios involving occlusion, semantic similarity, and low-saliency objects. The ablation studies confirm the contribution of each component: high-quality inpainting (LaMa), advanced MLM reasoning (GPT-5.2), and carefully designed prompts all significantly impact final ranking accuracy.

While our results validate the effectiveness of the proposed divide-and-conquer strategy, we also acknowledge the framework’s limitations, including its computational cost (O(n^2^) MLM inferences) and dependency on pre-trained component quality, which currently restricts its use to offline applications. Future work will focus on addressing these limitations by developing more efficient comparison strategies, exploring smaller and faster MLM variants suitable for edge deployment, and extending the pairwise comparison paradigm to other relative assessment tasks such as image aesthetic ranking and action importance ordering.

## Figures and Tables

**Table 1:** Comparative analysis of representative SOR approaches.

Method Category	Processing Paradigm	Relationship Modeling	Scalability (O(n) Complexity)	Generalization	Occlusion Robustness
End-to-End Global	Global, simultaneous	Implicit (via global features)	Limited (degrades as n increases)	Constrained by training set (fixed max n)	Implicit (learned from data)
Sequential	Sequential decomposition	Implicit (via attention mechanisms)	Moderate	Partially constrained	Implicit (learned from data)
Graph-Based	Global graph reasoning	Explicit (via graph structures on all objects)	Limited (graph complexity grows with n)	Constrained by training set	Implicit (via graph propagation)
PairwiseSOR-MLMs (Ours)	Local, pairwise decomposition	Explicit (via MLM comparisons on pairs)	High (O($n^{2}$) comparisons, but each is independent and scalable)	Training-free, zero-shot generalization	Explicit (via inpainting pre-processing)

**Table 2:** Quantitative comparison results of different methods.

Method	Reference	ASSR Testing Set			IRSR Testing Set		
		SA-SOR ↑	SOR ↑	MAE ↓	SA-SOR ↑	SOR ↑	MAE ↓
RSDNet	CVPR’18	0.631	0.775	0.123	0.423	0.710	0.117
ASSR	CVPR’20	0.657	0.792	0.101	0.415	0.652	0.109
SOR	ICCV’21	0.640	0.833	0.079	0.537	0.790	0.098
IRSR	TPAMI’21	0.703	0.828	0.091	0.565	0.814	0.095
OCOR	CVPR’22	0.646	0.873	0.102	0.507	0.814	0.100
PSR	ACMMM’23	0.636	0.828	0.075	0.454	0.752	0.080
QAGNet	CVPR’24	0.741	0.846	0.060	**0.610**	0.810	0.084
PoseSOR	ECCV’24	0.673	0.850	0.073	0.539	0.808	0.065
DSGNN	CVPR’24	0.723	0.843	0.066	0.568	0.785	0.073
SeqRank	AAAI’24	0.672	0.829	0.082	0.576	0.810	0.085
HyperSOR	TPAMI’24	0.653	0.830	0.101	-	-	-
LG-SOR	CVPR’25	0.733	0.882	0.065	0.578	0.817	0.060
Ours-limited	-	**0.750**	0.880	0.045	0.609	0.822	**0.053**
Ours-unlimited	-	0.746	**0.882**	**0.044**	0.603	**0.825**	**0.053**

Note: ↑ indicates that the larger the value, the better the indicator, and ↓ indicates that the smaller the value, the better the indicator; the bolded data indicate the best results; ”-” means no relevant data available.

**Table 3:** Ablation results of image inpainting model.

Model	IRSR Testing Set		
	SA-SOR ↑	SOR ↑	MAE ↓
MAT	0.600	0.811	0.060
TFill	0.603	0.818	0.062
LaMa	**0.609**	**0.822**	**0.053**

Note: ↑ indicates that the larger the value, the better the indicator, and ↓ indicates that the smaller the value, the better the indicator; the bolded data indicate the best results.

**Table 4:** Ablation results of large vision–language model.

Model	Parameters	IRSR Testing Set		
		SA-SOR ↑	SOR ↑	MAE ↓
InternVL3.5	~40B	0.585	0.808	0.069
Qwen3-VL	~7B/72B variants	0.599	0.810	0.063
LLaVA-v1.5-7B	~7B	0.590	0.807	0.067
GPT-5.2	Unknown (estimated >100B)	**0.609**	**0.822**	**0.053**

Note: ↑ indicates that the larger the value, the better the indicator, and ↓ indicates that the smaller the value, the better the indicator; the bolded data indicate the best results.

**Table 5:** Ablation result of the prompt.

Stage	Prompt	IRSR Testing Set		
		SA-SOR ↑	SOR ↑	MAE ↓
Pairwise Comparison	Full prompt: You are a visual saliency expert… Your task is to judge which object is more visually salient or attention-grabbing to a human observer…	**0.609**	**0.822**	**0.053**
	Simplified: Please indicate which object in the picture is more salient.	0.601	0.812	0.066
Global Ranking Aggregation	Full prompt: Given the following pairwise comparisons about which object is more visually salient, please infer a complete ranking list from most salient to least salient…	**0.609**	**0.822**	**0.053**
	Simplified: Please infer the overall sequence based on all the sorting results.	0.603	0.813	0.060

Note: ↑ indicates that the larger the value, the better the indicator, and ↓ indicates that the smaller the value, the better the indicator; the bolded data indicate the best results.

**Table 6:** Average inference time per image (in seconds) for different object counts.

Object Count (n)	Detection (YOLOv9)	Segmentation (SAM)	Inpainting (LaMa)	Pairwise Comparisons (n(n − 1)/2 MLM Calls)	Aggregation (1 MLM Call)	Total Time
3	0.12	0.28	0.35	3 × 1.2 = 3.6	1.5	5.85
5	0.12	0.45	0.58	10 × 1.2 = 12.0	1.5	14.65
8	0.12	0.72	0.92	28 × 1.2 = 33.6	1.5	36.86
10	0.12	0.91	1.15	45 × 1.2 = 54.0	1.5	57.68

Note: MLM API latency averages 1.2 s per call under stable network conditions, with minor variance (±0.3 s). Local processing (detection, segmentation, and inpainting) runs on GPU and scales approximately linearly with object count due to per-object mask processing.

**Table 7:** Frequency of logical conflicts.

Object Count	Total Triples	Conflicting Triples	Conflict Rate
3	1183	84	7.1%
4	2400	216	9.0%
5	1981	210	10.6%
6	1406	180	12.8%
7	1015	135	13.3%
8	600	90	15.0%
Overall	8585	915	10.7%

Note: Triples are weighted by frequency; counts are approximate for illustrative purposes.

**Table 8:** Comparison of aggregation methods.

Aggregation Method	SA-SOR ↑	SOR ↑	Cycle-Free Rankings
Bradley–Terry	0.592	0.805	82%
MLM-based (Ours)	0.609	0.822	96%

Note: ↑ indicates that the larger the value, the better the indicator.

## Data Availability

The data presented in this study are available upon request from the author.

## Abbreviations

The following abbreviations are used in this manuscript:

SOR	Salient Object Ranking
MLMs	Multimodal Large Models
SOD	Salient Object Detection
SA-SOR	Segmentation-Aware Salient Object Ranking
MAE	Mean Absolute Error
ISSR	Image Segmentation and Scene Reconstruction

## References

[1] Zhang L., Zhang J., Lin Z., Lu H., He Y. Capsal: Leveraging captioning to boost semantics for salient object detection. Proceedings of the IEEE/CVF Conference on Computer Vision and Pattern Recognition.

[2] Wu Y., Liu Y., Zhang L., Cheng M., Ren B. (2022). Edn: Salient object detection via extremely-downsampled network. IEEE Trans. Image Process..

[3] Liu J., Hou Q., Liu Z., Cheng M. (2022). Poolnet+: Exploring the potential of pooling for salient object detection. IEEE Trans. Pattern Anal. Mach. Intell..

[4] Zhang Q., Xiao X., Wang X., Wang S., Kwong S., Jiang J. (2022). Adaptive viewpoint feature enhancement-based binocular stereoscopic image saliency detection. IEEE Trans. Circuits Syst. Video Technol..

[5] Song X., Guo F., Zhang L., Lu X., Hei X. (2024). Salient object detection with dual-branch stepwise feature fusion and edge refinement. IEEE Trans. Circuits Syst. Video Technol..

[6] Islam M., Kalash M., Bruce N. Revisiting salient object detection: Simultaneous detection, ranking, and subitizing of multiple salient objects. Proceedings of the IEEE Conference on Computer Vision and Pattern Recognition.

[7] Du L., Li L., Wei D., Mao J. (2019). Saliency-guided single shot multibox detector for target detection in sar images. IEEE Trans. Geosci. Remote Sens..

[8] Li H., Zhang D., Liu N., Cheng L., Dai Y., Zhang C., Wang X., Han J. Boosting low-data instance segmentation by unsupervised pre-training with saliency prompt. Proceedings of the IEEE/CVF Conference on Computer Vision and Pattern Recognition.

[9] Miangoleh S., Bylinskii Z., Kee E., Shechtman E., Aksoy Y. Realistic saliency guided image enhancement. Proceedings of the IEEE/CVF Conference on Computer Vision and Pattern Recognition.

[10] Itti L., Koch C., Niebur E. (2022). A model of saliency-based visual attention for rapid scene analysis. IEEE Trans. Pattern Anal. Mach. Intell..

[11] Bruce N., Tsotsos J. (2005). Saliency based on information maximization. Adv. Neural Inf. Process. Syst..

[12] Walther D., Koch C. (2006). Modeling attention to salient proto-objects. Neural Netw..

[13] Liu N., Li L., Zhao W., Han J., Shao L. (2021). Instance-level relative saliency ranking with graph reasoning. IEEE Trans. Pattern Anal. Mach. Intell..

[14] OpenAI Gpt-4v(ision) System Card. 2023, Technical Report. https://cdn.openai.com/papers/GPTV_System_Card.pdf.

[15] Liu H., Li C., Wu Q., Lee Y. (2023). Visual instruction tuning. Adv. Neural Inf. Process. Syst..

[16] Bai J., Bai S., Yang S., Wang S., Tan S., Wang P., Lin J., Zhou C., Zhou J. (2023). Qwen-vl: A versatile vision-language model for un derstanding, localization, text reading, and beyond. arXiv.

[17] Cerf M., Frady E., Koch C. (2009). Faces and text attract gaze independent of the task: Experimental data and computer model. J. Vis..

[18] Borji A., Itti L. Exploiting local and global patch rarities for saliency detection. Proceedings of the IEEE/CVF Conference on Computer Vision and Pattern Recognition.

[19] Cheng M., Mitra N., Huang X., Torr P., Hu S. (2014). Global contrast based salient region detection. IEEE Trans. Pattern Anal. Mach. Intell..

[20] Zeng Z., Liu H., Chen F., Tan X. (2023). Airsod: A lightweight network for rgb-d salient object detection. IEEE Trans. Circuits Syst. Video Technol..

[21] Li A., Mao Y., Zhang J., Dai Y. (2023). Mutual information regularization for weakly-supervised rgb-d salient object detection. IEEE Trans. Circuits Syst. Video Technol..

[22] Cong R., Qin Q., Zhang C., Jiang Q., Wang S., Zhao Y., Kwong S. (2023). A weakly supervised learning framework for salient object detection via hybrid labels. IEEE Trans. Circuits Syst. Video Technol..

[23] Wang K., Tu Z., Li C., Zhang C., Luo B. (2024). Learning adaptive fusion bank for multi-modal salient object detection. IEEE Trans. Circuits Syst. Video Technol..

[24] Tang B., Liu Z., Tan Y., He Q. (2023). Hrtransnet: Hrformer-driven two modality salient object detection. IEEE Trans. Circuits Syst. Video Technol..

[25] Siris A., Jiao J., Tam G., Xie X., Lau R. Inferring attention shift ranks of objects for image saliency. Proceedings of the IEEE/CVF Conference on Computer Vision and Pattern Recognition.

[26] Fang H., Zhang D., Zhang Y., Chen M., Li J., Hu Y., Cai D., He X. Salient object ranking with position-preserved attention. Proceedings of the IEEE/CVF International Conference on Computer Vision.

[27] Tian X., Xu K., Yang X., Du L., Yin B., Lau R. Bi-directional object-context prioritization learning for saliency ranking. Proceedings of the IEEE/CVF Conference on Computer Vision and Pattern Recognition.

[28] Guan H., Lau R. Seqrank: Sequential ranking of salient objects. Proceedings of the AAAI Conference on Artificial Intelligence.

[29] Qiao M., Xu M., Jiang L., Lei P., Wen S., Chen Y., Sigal L. (2024). Hypersor: Context-aware graph hypernetwork for salient object ranking. IEEE Trans. Pattern Anal. Mach. Intell..

[30] Wu Z., Lu J., Han J., Bai L., Zhang Y., Zhao Z., Song S. Domain separation graph neural networks for saliency object ranking. Proceedings of the IEEE/CVF Conference on Computer Vision and Pattern Recognition.

[31] Deng B., Song S., French A., Schluppeck D., Pound M. Advancing saliency ranking with human fixations: Dataset models and benchmarks. Proceedings of the IEEE/CVF Conference on Computer Vision and Pattern Recognition.

[32] Hou R., Li X., Ren T., Zhou D., Wu G., Cao J. (2025). HyPSAM: Hybrid Prompt-Driven Segment Anything Model for RGB-Thermal Salient Object Detection. IEEE Trans. Circuits Syst. Video Technol..

[33] Zhang S., Huang J., Tang W., Wu Y., Hu T., Xu X., Liu J. DiMSOD: A Diffusion-Based Framework for Multi-Modal Salient Object Detection. Proceedings of the AAAI Conference on Artificial Intelligence.

[34] Tang H., Li Z., Zhang D., He S., Tang J. (2024). Divide-and-conquer: Confluent triple-flow network for RGB-T salient object detection. IEEE Trans. Pattern Anal. Mach. Intell..

[35] Wang C., Yeh I., Mark Liao H. (2024). Yolov9: Learning what you want to learn using programmable gradient Information. Proceedings of the European Conference on Computer Vision, Milan, Italy, 29 September–4 October 2024.

[36] Suvorov R., Logacheva E., Mashikhin A., Remizova A., Ashukha A., Silvestrov A., Kong N., Goka H., Park K., Lempitsky V. Resolution-robust large mask inpainting with fourier convolutions. Proceedings of the IEEE/CVF Winter Conference on Applications of Computer Vision.

[37] Lin T., Maire M., Belongie S., Hays J., Perona P., Ramanan D., Doll’ar P., Zitnick C. (2014). Microsoft coco: Common objects in context. Proceedings of the Computer Vision–ECCV 2014: 13th European Conference, Zurich, Switzerland, 6–12 September 2014.

[38] Jiang M., Huang S., Duan J., Zhao Q. Salicon: Saliency in context. Proceedings of the IEEE Conference on Computer Vision and Pattern Recognition.

[39] Sun C., Xu Y., Pei J., Fang H., Tang H. Partitioned saliency ranking with dense pyramid transformers. Proceedings of the 31st ACM International Conference on Multimedia.

[40] Guan H., Lau R. (2024). Posesor: Human pose can guide our attention. Proceedings of the European Conference on Computer Vision, Milan, Italy, 29 September–4 October 2024.

[41] Liu F., Liu Y., Xu K. Language-Guided Salient Object Ranking. Proceedings of the IEEE/CVF Conference on Computer Vision and Pattern Recognition.

[42] Zheng C., Cham T., Cai J., Phung D. Bridging global context interactions for high-fidelity image completion. Proceedings of the IEEE/CVF Conference on Computer Vision and Pattern Recognition.

[43] Li W., Lin Z., Zhou K., Qi L., Wang Y., Jia J. Mat: Mask-aware transformer for large hole image inpainting. Proceedings of the IEEE/CVF Conference on Computer Vision and Pattern Recognition.

[44] Wang W., Gao Z., Gu L., Pu H., Cui L., Wei X., Liu Z., Jing L., Ye S., Shao J. (2025). Internvl3.5: Advancing open-source multimodal models in versatility, reasoning, and efficiency. arXiv.

